# Murine skin-derived multipotent papillary dermal fibroblast progenitors show germline potential in vitro

**DOI:** 10.1186/s13287-023-03243-5

**Published:** 2023-02-03

**Authors:** Wei Ge, Yuan-Chao Sun, Tian Qiao, Hai-Xia Liu, Tao-Ran He, Jun-Jie Wang, Chun-Lei Chen, Shun-Feng Cheng, Paul W. Dyce, Massimo De Felici, Wei Shen

**Affiliations:** 1 College of Life Sciences, Key Laboratory of Animal Reproduction and Biotechnology in Universities of Shandong, Qingdao Agricultural University, Qingdao, 266109 China; 2 Department of Animal Sciences, Auburn University, Auburn, AL 36849 USA; 3 Department of Biomedicine and Prevention, University of Rome Tor Vergata, 00133 Rome, Italy

**Keywords:** Skin-derived stem cells, Papillary dermal fibroblast progenitors, Single-cell transcriptomes, Spermatogonial stem cells like cells

## Abstract

**Background:**

Many laboratories have described the in vitro isolation of multipotent cells with stem cell properties from the skin of various species termed skin-derived stem cells (SDSCs). However, the cellular origin of these cells and their capability to give rise, among various cell types, to male germ cells, remain largely unexplored.

**Methods:**

SDSCs were isolated from newborn mice skin, and then differentiated into primordial germ cell-like cells (PGCLCs) in vitro. Single-cell RNA sequencing (scRNA-seq) was then applied to dissect the cellular origin of SDSCs using cells isolated from newborn mouse skin and SDSC colonies. Based on an optimized culture strategy, we successfully generated spermatogonial stem cell-like cells (SSCLCs) in vitro.

**Results:**

Here, using scRNA-seq and analyzing the profile of 7543 single-cell transcriptomes from newborn mouse skin and SDSCs, we discovered that they mainly consist of multipotent papillary dermal fibroblast progenitors (pDFPs) residing in the dermal layer. Moreover, we found that epidermal growth factor (EGF) signaling is pivotal for the capability of these progenitors to proliferate and form large colonies in vitro. Finally, we optimized the protocol to efficiently generate PGCLCs from SDSCs. Furthermore, PGCLCs were induced into SSCLCs and these SSCLCs showed meiotic potential when cultured with testicular organoids.

**Conclusions:**

Our findings here identify pDFPs as SDSCs derived from newborn skin and show for the first time that such precursors can be induced to generate cells of the male germline.

**Supplementary Information:**

The online version contains supplementary material available at 10.1186/s13287-023-03243-5.

## Introduction

Over the past few decades, scientists have explored whether functional gametes can be obtained from many different types of stem cells under in vitro conditions [1, 2]. Recently, complete spermatogenesis and oogenesis under in vitro conditions have been established from mouse pluripotent embryonic stem cells (ESCs) or induced pluripotent stem cells (iPSCs) [3, 4]. As far as we know, it has not been possible to reproduce these results starting from adult stem cells (ASCs), although some types of ASCs were shown to be capable of generating germ cell-like cells (GCLCs) in vitro [5–10].

In 2001, skin-derived precursors representing novel multipotent adult stem cells, distinct from epidermal stem cells (EPSCs) and dermal mesenchymal stem cells (MSCs), were isolated from juvenile and adult mice [11]. These cells, possibly derived from neural crest or mesodermal cells, were reported to reside in the dermis and be able to generate cells of different lineages, such as glia, adipocytes, neuron, and smooth muscle cells. Cells with similar characteristics termed skin-derived stem cells (SDSCs), were isolated also from other species including pigs and humans [12–14]. In 2006, Dyce and colleagues reported the generation of oocyte-like cells (OLCs) from fetal porcine SDSCs [8]. Subsequent studies demonstrated that SDSCs with germ cell potential could be generated from neonatal mouse skin [7, 15, 16]. Likewise, female and male human fetal SDSCs were differentiated into germ cell-like cells (GCLCs) able to initiate defective meiosis in vitro [17]. Intriguingly, aggregates consisting of OLCs and ovarian somatic cells derived from mouse SDSCs were able to secrete estradiol and progesterone in response to gonadotropin stimulation and to restore endogenous serum estradiol levels when transplanted in ovariectomized females [16]. Such OLCs were generated from CD34-positive hair follicle stem cells (HFSCs) isolated from early postnatal mice [6]. However, the derivation of GCLCs from SDSCs remains an inefficient process restricting its applicative possibilities. In addition, the cellular origin of SDSCs generated from neonatal skin remains elusive.

Using single-cell RNA sequencing (scRNA-seq) we recently analyzed cell fate commitment during in utero skin development in mice and goats [18, 19]. A single-cell transcriptome atlas of neonatal mouse skin is now available providing a reference for understanding cellular heterogeneity of SDSCs cultured in vitro.

In the present study, we use scRNA-seq to dissect the cellular heterogeneity of SDSCs generated in vitro from neonatal skin and unveil their cellular origin from multipotent papillary dermal fibroblast progenitors (pDFPs). Utilizing a modified protocol, adapted from those described to induce functional gametes from mouse pluripotent stem cells, we show that these cells could be efficiently induced to give rise to primordial germ cell-like cells (PGCLCs) in vitro. Furthermore, PGCLCs can be induced into spermatogonia stem cell-like cells (SSCLCs) in vitro and these SSCLCs showed meiotic potential when cultured with testicular organoids.

## Materials and methods

### Experimental animals

All animals used in the current study were housed and maintained in a temperature (22–24 °C) and light (12 h light and 12 h dark) controlled room with ad libitum access to food and water. CAG/eGFP transgenic mice (Stock TgN (GFPU) 5 Nagy) strain with ubiquitously expressed eGFP (kindly provided by Dr. Xiao Yang from the Institute of Biotechnology, Beijing), were used for monitoring SSCLCs differentiated from the SDSCs-generated PGCLCs in reconstituted testicular organoids. CD-1 mice were used for MEF feeder cell preparation and testicular somatic cell isolation and were purchased from the Vital River Laboratory Animal Technology Co., Ltd, Beijing. Mice were killed by decapitation and about 120 CAG/eGFP transgenic mice and 10 CD-1 mice were involved in the current study. Experimental animal management was approved by the Ethics Committee of Qingdao Agricultural University (Approval Number: 2019-021).

### Dissociation of skin and generation of SDSC spheres

The skin dissociation procedure used here is described in detail in [7]. Briefly, the dorsal skin of newborn CAG/eGFP transgene male mice was isolated using surgical scissors following euthanasia. After removing the fat tissue and contaminating blood with forceps, the tissues were cut into 1 mm^2^ pieces with scissors. Dissociation was then performed with trypsin–EDTA (0.25%) solution (Sorlabio, T1300, Beijing, China) by incubating for 25 min at 37 °C. After dissociation, cells were dispersed by mechanical pipetting, and a single-cell suspension was obtained using a cell strainer (40 μm, Biologix, 15-1040, Grand Island, NY, USA). The obtained single cells were then cultured in DMEM/F12 (Thermo Fisher Scientific, 8121453, Beijing, China) medium containing 40 ng/ml basic fibroblast growth factor (bFGF, PeproTech, 100-18B, Rocky Hill, NJ, USA), 1 × B-27 (Gibco, 17504044, Grand Island, NY, USA), 20 ng/ml epidermal growth factor (EGF, R&D Systems, 2028-EG, Minneapolis, MN, USA), and 1% penicillin/streptomycin (Solarbio, P1400, Beijing, China) in culture dishes for cells growing in suspension (Sarstedt, 83.3902.500, Nümbrecht, Germany) at 37 °C, 5% CO_2_. Medium was changed every four days and the generated SDSC spheres were harvested after 12 days of culture for further procedures.

### Preparation of MEF feeder cells

Mitotically inactivated mouse embryonic fibroblast (MEF) feeder cells were obtained following standard procedures as described in *Curr. Protoc. Stem Cell Biol.* 3:1C.3.1-1C.3.17. © 2007 by John Wiley & Sons, Inc.

### Generation of PGCLCs

A two-step procedure was used to generate PGCLCs from SDSCs. First, in order to produce epiblast cell like cells (EpiLCs), P2 SDSC spheres were mechanically dispersed into a single-cell suspension and seeded at 5 × 10^5^ cells/well into a 24-well plate for cells growing in suspension. The EpiLC induction medium was M199 supplemented with 0.5% insulin-transferrin-selenium (ITS, Thermo Fisher Scientific, 41400045, Waltham, MA, USA), 0.1% bovine fetuin (Merck Millipore, 341506, Palmerston North, New Zealand), 1.5% bovine serum albumin (BSA, Sigma, B2064), 30 ng/ml BMP4 (R&D Systems, 314-BP-050), 0.23 mM sodium pyruvate (Gibco, 11360-070), 1 ng/ml EGF in media medium 199 (Thermo Fisher Scientific, 11150067, Beijing, China). The medium was changed every two days and the first EpiLC colonies were observed after 12 h of culture. In the second step, to induce PGCLCs, colonies of EpiLCs on day 4 were dissociated into single cells by pipetting and plated onto mitotically inactivated MEF feeder cells at a concentration of 5 × 10^5^ cells/ml. The PGCLC differentiation medium was DMEM/high glucose (Gibco, C11995500BT), supplemented with 10% FBS, 40 ng/ml bFGF, 40 ng/ml SCF (R&D Systems, 455-MC-010), 20 ng/ml EGF, 1% MEM non-essential amino acids solution (Thermo Fisher Scientific, 11140-050, Waltham, MA, USA), 1% GlutaMAX™ Supplement (Thermo Fisher Scientific, 35050061, Waltham, MA, USA), 1% sodium pyruvate, 50 μM β-mercaptoethanol (Sigma, M-7522), and 1% penicillin/streptomycin. Half medium was changed every two days being careful not to remove suspended cells.

### Differentiation of SSCLCs from PGCLCs

For SSCLCs differentiation from PGCLCs, we used a testicular organoid culture system termed the three-layer gradient system previously described [20] with some modifications. Briefly, testes dissected from 7 days postpartum CD-1 mice were cut into small pieces and a monodispersed cell population was obtained by subsequent incubation in 2 mg/ml collagenase IV solution (Sigma, C5138) for 15 min and TrypLE™ Express enzyme for 10 min at 37 °C, followed by repeated pipetting in DMEM. Cells were then resuspended in SSCLC induction medium and plated into a 10 cm tissue culture dish treated with 0.2% (w/v) gelatin. Not adherent cells (mainly germ cells) were removed through aspirating the supernatant after 4, 12, and 24 h in vitro culture while the somatic cells attached at the bottom of the culture dish were harvested at 24 h and combined with 8–10 day induced eGFP-PGCLCs (cell ratio 1:1) at a concentration of 4 × 10^7^ cells/ml. The cell suspension was then mixed with Matrigel (Corning, 356231) at a ratio of 1:1 and about 16 μl of this mixture was transferred onto a layer of 1.5% agarose gel (Tsingke Biotechnology, TSJ001, Beijing, China) applied to the bottom of a 24-well plate. The plate was transferred to the incubator for 15 min at 37 °C for gelification. Finally, 400 μl of SSC induction medium was added to the well and the culture was carried out in a 5% CO_2_ humidified incubator at 37 °C. The SSC induction medium was referenced from Ishikura et al. [21] and consisted of StemPro-34 SFM medium (Gibco, 10640-019) supplemented with 20 ng/ml EGF, 1 × insulin-transferrin-selenium, 10 ng/ml bFGF, 1 μl/ml DL-lactic acid (Sigma, 69785), 2 mM L-glutamine, 6 mg/ml d-( +)-glucose (Sigma, G8270), 30 μg/ml pyruvic acid (Sigma, 107360), 5 mg/ml BSA, 60 ng/ml progesterone (Sigma, P0130), 1 × MEM vitamin solution (Gibco, 11120-052), 1 × non-essential amino acids, 30 ng/ml β-estradiol (Sigma, E8875), 50 μM β-mercaptoethanol, 100 nM ascorbic acid (Sigma, A4544), 1% FBS, 10 μg/ml d-biotin (Sigma, B-3399), and 10 ng/ml glial cell line-derived neurotrophic factor (GDNF, R&D Systems, 212-GD-050).

### Immunofluorescence and immunohistochemistry

For immunofluorescence (IF), cell clusters or dissociated cells were fixed with 4% paraformaldehyde (Sorlabio, P1110) on ice for 30 min. Then, the cells were transferred to glass slides for drying. Samples were then permeabilized with 0.5% Triton X-100 in PBS (Sorlarbio, T8200) for 10 min, and incubated with blocking buffer (permeabilization containing 10% goat blocking serum, BOSTER, AR0009, Wuhan, China) for 30 min. After blocking, the samples were then incubated with the corresponding primary antibodies overnight at 4 °C. Secondary antibodies were added according to the species source of primary antibodies incubation carried out at 37 °C for 2 h. Vectashield mounting media (Vector Laboratories, H-1000, Burlingame, CA, USA) for mounting and a confocal microscope (Leica, TCS SP5 II, Wetzlar, Germany) for image acquisition were used at 20x. Immunohistochemistry (IHC) of skin sections was performed using the procedures we recently described [18]. All antibodies used are listed in Additional file 1: Table S1.

### Bisulfite sequencing

Bisulfite sequencing was performed as previously described [22, 23]. Briefly, the TIANGEN Micro DNA isolation Kit (Tiangen, DP316, Beijing, China) was used to extract genomic DNA from PGCLCs. Bisulfite treatment was then performed using an EpiTect® Bisulfite kit (EpiTect, 59104, Hilden, Germany). Differentially methylated regions of *Igf2r* and *Peg3* genes were then amplified by RT-PCR as previously described [24] and the target sequences cloned into the pDM19-T Vector (TaKaRa, D102A, Dalian, China) for downstream sequencing.

### Gene expression analysis

Total RNA was extracted from cell aggregates using SPARKeasy Tissue/Cell RNA Rapid Extraction Kit (SPARKjade, AC0202, Shandong, China) and reverse transcription was performed using a SPARKScript RT plus Kit (SPARKjade, AG0304). A Light Cycler SYBR Green I Master (Roche, 04707516001, Mannheim, Germany) kit was used to prepare PCR mix and PCR was performed using a Light Cycler 480 Real-Time PCR System (Roche, Mannheim, Germany). The relative gene expression was calculated using the 2^−(△△Ct)^ method and *Gaphd* was used as a housekeeping gene. Primer sequences used are listed in Additional file 2: Table S2.

### Western blotting analysis

Cells were firstly harvested by centrifugation and lysed using RIPA lysis solution (Beyotime, P0013B, Beijing, China). The extracts were then analyzed by agarose gel electrophoresis with 12% SDS-PAGE, at 80 V for 20 min, and at 120 V for 2.5 h. Bio-Rad Trans-Blot (Bio-Rad, Hercules, California, USA) was used to transfer the proteins onto the polyvinylidene difluoride membranes (Millipore, ISEQ00010, Bedford, MA, USA). Blocking was performed with PBST containing 5% BSA on ice for 4 h and the membranes were incubated with primary and secondary antibodies for 4 h and overnight, respectively. BeyoECL Star Chemiluminescence Kit (Beyotime, P0018S, Beijing, China) was used for protein band detection. Quantification of protein blots was performed using AlphaView SA software (Alpha Innotech Corporation, San Leandro, CA, USA) according to the user guide using default parameters.

### Meiotic chromosome spread

Meiotic chromosome spread staining was performed as we previously described [25]. Briefly, testicular organoid tissues were harvested with a 1 ml pipette and transferred in a hypotonic solution containing 30 mM Tris (Sorlabio, T8060), 50 mM sucrose (Sorlabio, S8271), 17 mM citric acid (Sorlabio, C8610), 5 mM EDTA (Sorlabio, E8040), 2.5 mM dl-dithiothreitol (Sorlabio, D8220) and 1 mM phenylmethanesulfonyl fluoride (Sorlabio, IP0280) in deionized water for 45 min on ice. The samples were then transferred to a 4% paraformaldehyde solution and were dispersed using a pair of precision forceps before spreading evenly on a glass slide. After overnight at room temperature, the slides were washed with a wetting agent (Kodak, 1464502, Rochester, NY, USA) and then treated with the blocking buffer containing 1% goat serum in PBS for 1 h at room temperature. The samples were further incubated with the corresponding primary antibodies at 37 °C overnight. After careful washing, the matched secondary antibodies were added, and incubation was carried out at 37 °C for 2 h. At last, the slides were incubated in Hoechst 33342 (Beyotime, C1017) or DAPI (Beyotime, C1002) solution to stain nuclei for 5 min at room temperature.

### Single-cell cDNA library preparation and sequencing

We used 10 × Genomics’ Chromium Single Cell 3' V3 Gel Beads Kit (10 × Genomics, PN-1000075, Pleasanton, CA, USA) to construct a single-cell cDNA library, and all procedures were performed according to the recommended protocol. Briefly, cells with cell viability higher than 90% were projected for cell capture using a 10 × Genomics Chromium controller (10 × Genomics, Pleasanton, CA). Sequencing was performed by Novogene, Inc.

### 10 × Genomics scRNA-seq data preprocessing

Alignment and quantification of UMI counts were performed using the CellRanger (v3.0.2) software according to the standard protocol (https://www.10xgenomics.com/). After that, the processed expression matrix was then analyzed using the Seurat (v3) R package for downstream quality control and visualization [26]. For quality control, cells were filtered out using the default parameter in Seurat and data integration was performed with *FindIntegrationAnchors* function in the Seurat package. Dimension reduction and cluster identification were performed with *RunUMAP* and *FindClusters* functions with the following parameters: dims = 1:25, resolution = 0.8. Other parameters were set as default according to the official instructions.

### RNA velocity analysis and single-cell trajectory

To determine the directionality of differentiation, the *velocyto* package was used to infer velocity vectors in single cells according to the published protocol [27]. Briefly, the spliced and unspliced count metrics were firstly generated directly from the Cellranger output file using *velocyto run10x* function using the standard procedure. The obtained metric files were further analyzed using *scVelo* package [28], and the velocities were projected onto pre-computed UMAP embedding using *scv.pl.velocity_embedding_stream* function with default parameters.

For single-cell trajectory inference, the slingshot R package was used to infer dynamic gene expression along pseudotime [29]. The root cell cluster was determined according to the directionality of RNA velocity vectors incorporated in the UMAP plot as previously described [30].

### Gene ontology (GO) enrichment

We used Metascape to perform GO enrichment analysis and the analysis procedure was performed according to the online tutorial (https://metascape.org/) [31].

### Statistical analysis

Statistical tests were analyzed using the Graphpad Prism 7 software. Statistical significance was analyzed with the Student’s unpaired t-test or one-way ANOVA test.

## Results

### Dissection of cellular heterogeneity of P2 SDSCs and newborn mouse skin at single-cell resolution

All SDSCs described in the current study were isolated from newborn male pup skin according to published protocols [7, 8, 32]. Transgenic mice carrying a ubiquitously expressed enhanced green fluorescent protein (CAG/eGFP) were used to generate SDSCs in order to obtain traceable cells for the experiments involving the culture of SSCLCs within testicular organoids (Additional file 3: Fig. S1A).

**Fig. 1:**
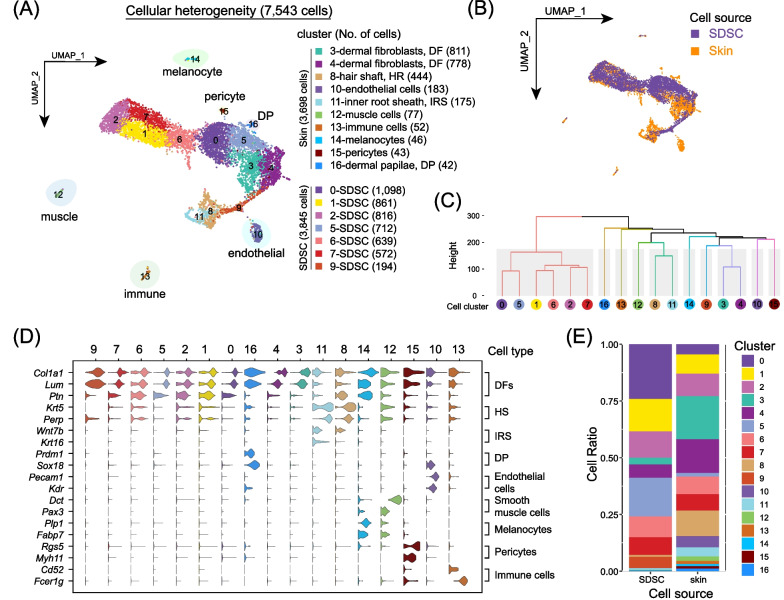
Analysis of single-cell transcriptome profiles of newborn mouse skin and P2 SDSCs. **A** UMAP projection of 7543 single-cell profiles reveals 17 cell clusters in newborn skin and SDSCs. **B** UMAP plot color-coded by cell source. **C** Hierarchical clustering of different cell clusters identified by UMAP analysis. **D** Representative canonical cell type-specific marker expression across all cell clusters. **E** Percentage of different cell clusters in newborn skin and SDSCs

scRNA-seq was used to analyze the cellular heterogeneity in newborn skin and SDSCs cultured to passage two (P2). We generated 8000 single-cell transcriptome profiles in total (Additional file 3: Fig. S1B), and after rigorous quality control (Additional file 3: Fig. S1C), 7543 single-cell transcriptome profiles were subjected to transformations into low-dimensional spaces by uniform manifold approximation and projection (UMAP). After batch correlation, we identified a total of 17 cell clusters of which 7 were mainly comprised of cell populations derived from SDSCs (Fig. 1A, B). Hierarchical clustering indicated that 6 of these, namely 0, 1, 2, 5, 6, and 7, clustered in the same branch consistent with their origin from SDSCs, while cluster 9 clustered with the other skin-derived cell types (Fig. 1C).

To further define the cell identities in the UMAP plot we annotated the different cell clusters using a series of canonical marker genes [18]. On the basis of the higher expressed genes, nine different cell types were identified as follows: SDSC clusters 0, 1, 2, 5, 6, 7 and 9 showing high expression of the dermal cell lineage gene markers *Ptn*, *Col1a1* and *Lum* were designated as dermal fibroblast (DF) populations, cluster 8 (*Krt5* and *Perp*) as hair shaft cells, cluster 10 (*Pecam1* and *Kdr*) as endothelial cells, cluster 11 (*Wnt7b* and *Krt16)* as inner root sheath cells (IRS), cluster 12 (*Dct* and *Pax3*) as muscle cells, cluster 13 (*Cd52* and *Fcer1g)* as immune cells, cluster 14 (*Plp1* and *Fabp7*) as melanocytes, cluster 15 (*Rgs5* and *Myh11)* as pericytes and cluster 16 expressing *Prdm1* and *Sox18,* as dermal papillae cells (DP) (Fig. 1D and Additional file 3: Fig. S1D). The comparison of the cluster composition between newborn mouse skin tissues and P2 SDSCs indicated that SDSCs cells were heterogeneous, and mainly consisted of dermal lineage-derived cells (Fig. 1E).

### Reconstruction of cellular differentiation trajectory and RNA velocity analysis unveil the origin of SDSCs

**Fig. 2:**
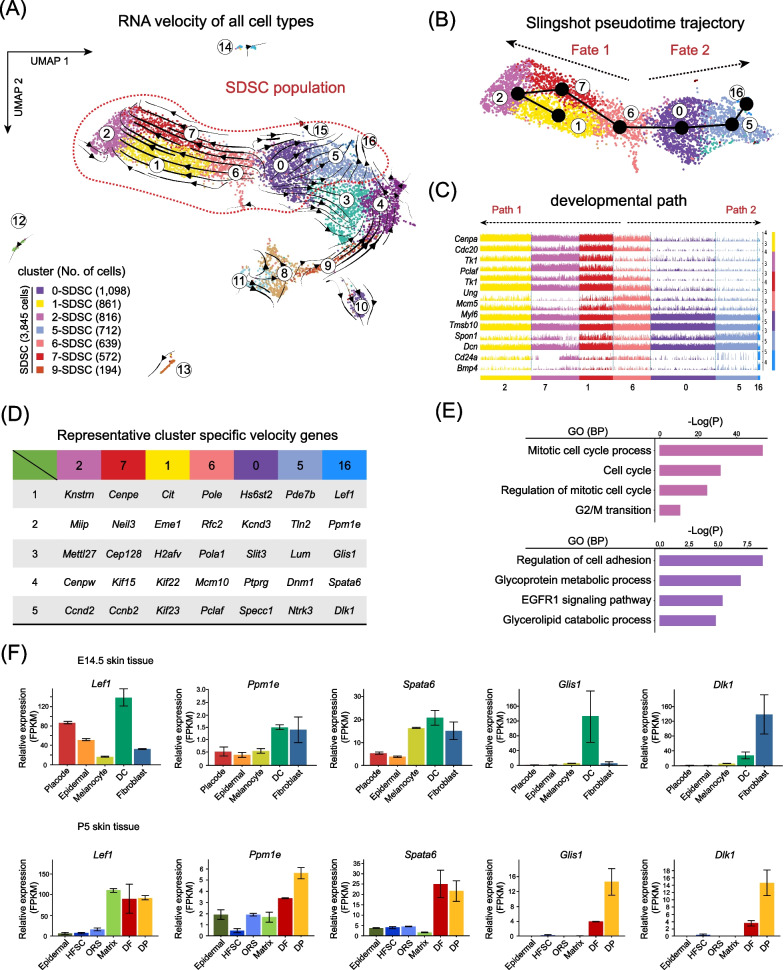
Combined RNA velocity and trajectory inference unveil the cellular origin of SDSCs. **A** Projection of RNA velocity vectors in the UMAP plot. **B** Slingshot infers the pseudotime trajectory in the P2 SDSCs. **C** Expression of cell fate 1 and cell fate 2 representative marker genes along pseudotime trajectories. **D** Expression of top 5 cell cluster-specific RNA velocity genes; genes were ranked by their roles in driving the velocity trajectories. **E** GO enrichment analysis of top 100 RNA velocity genes in the end state of cell fate 1 and 2. **F** Expression of top 5 cell fate 2 RNA velocity genes in the skin of E14.5 foetuses and 5 dpp newborn skin

Since it is known that in the neonatal skin reside dermal fibroblasts (DFs) with distinct differentiation potential, including papillary, reticular and hypodermal fibroblasts [33], we next investigated DF lineages ongoing in the in vitro cultured SDSCs. In order to capture the differentiation trajectories of DFs, we utilized RNA velocity analysis that is able to predict cell fate on the basis of the ratio in scRNA data between spliced and un-spliced mRNA and is considered a robust tool to identify cell fate commitment in heterogeneous single cell RNA-seq data [27]. Noteworthy, we found that the projection of RNA velocity vectors onto UMAP plots showed two distinct differentiation directories (Fig. 2A). Clusters 0 and 6 were root states, hence representing cells at the beginning of differentiation [30], while clusters 2 and 16 represented final differentiation states. From the root states, we reconstructed two pseudotime differentiation trajectories towards the end states to identify gene expression dynamics of cell fates 1 (cluster 2) and 2 (cluster 16). The results showed that cells at final fate state 1 (cluster 1, 2, 6, 7) expressed high levels of *Cenpa* and *Cdc20,* genes involved in mitotic cell cycle processes, while the dermal papillae marker genes *Lef1* and *Ppm1e* were expressed at higher levels by cells at the final fate of state 2 (cluster 0, 5, 16) [34] (Fig. 2B, C). We further identified RNA velocity driver genes for both differentiation trajectories (Fig. 2D). For cell fate 1, the top enriched RNA velocity driving genes, ranked by their roles in driving the velocity trajectories [34], included *Knstrn* which encodes a protein involved in chromosome segregation during mitosis [35], and *Miip* which encodes a negative regulator of cell migration and mitosis [36]. Moreover, gene ontology (GO) enrichment analysis of the top 100 RNA velocity driver genes showed that “mitotic cell cycle process”, “cell cycle”, and “regulation of mitotic cell cycle”, were the top represented GO terms for cell fate 1 (Fig. 2E). In line with this, heatmap of cluster-specific represented GO terms and a comparison of shared genes and GO identified the expression of cell cycle mitotic genes along this pathway (Additional file 3: Fig. S2A, B). Interestingly, for cell fate 2 (cluster 16) top-ranked RNA velocity genes resulted the same *Lef1*, the dermal papillae cell fate RNA velocity driving gene identified previously in vivo [34], and *Ppm1e*, encoding a regulator of AMPK phosphorylation [37]. On this basis, we designated fate 1 as a cell renewal state and fate 2 as the differentiation into the papillary fibroblast lineage. Collectively, these data demonstrated that SDSCs mainly consist of papillary dermal fibroblast progenitors (pDFPs) able to self-renewal and differentiation into papillary DFs in vitro.

To further validate our RNA velocity analysis, we retrieved mRNA expression data from Rezza et al. [38] and assessed the expression of the top 5 RNA velocity genes in the endpoint of cell fate 2 (*Lefty*, *Ppm1e*, *Spata6*, *Glis1*, *Dlk1*) in the skin of E14.5 embryos and 5 dpp pups (Fig. 2F) [39]. Such analysis established that these genes were mainly expressed in the E14.5 skin dermal cells (DCs) and showed higher expression in the DF and DP cells in the 5 dpp pup skin. This validated our RNA velocity analysis and confirmed that SDSCs (hereafter referred to as pDFPs) mainly comprise papillary dermal fibroblast progenitors able to differentiate into DP cells [33].

### EGF promotes pDFP proliferation in vitro

**Fig. 3:**
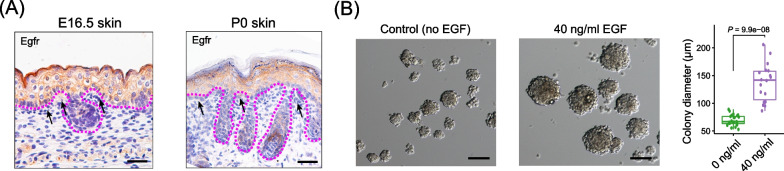
EGF promotes proliferation and formation of large pDFPs spheres (colonies) in vitro. **A** IF staining of EGFR in epidermal cells, hair follicles and dermal fibroblasts of the skin of E16.5 fetuses and 0 dpp pups; dotted lines indicate dermal and epidermal interface, and black arrows indicate papillary dermis. Pictures were taken using an optical microscope (20x, Olympus BX51, Japan). Scale bars, 100 μm. **B** Effects of EGF on the diameter of P2 pDFP colonies. Pictures were taken using an eclipse microscope (10x, Nikon TE2000, Japan)

Since the enriched GO terms of cell fate 2 RNA velocity driving genes analysis also included the “EGFR1 signaling pathway”, we then tested if EGFR signaling was active and has a role in the cultured pDFPs. We first confirmed by immunohistochemistry that EGFR was expressed in the skin of E16.5 fetuses and 0 dpp pups in epidermal cells, hair follicles, and dermal fibroblasts (Fig. 3A). Next removing EGF from the culture medium, we found that the ability of pDFPs to generate large colonies was significantly reduced (Fig. 3B). Collectively, these results further confirmed that SDSCs consist of multipotent pDFPs, and EGF signaling is pivotal for pDFPs colony formation in vitro.

### Specification of PGCLCs from pDFPs in vitro

**Fig. 4:**
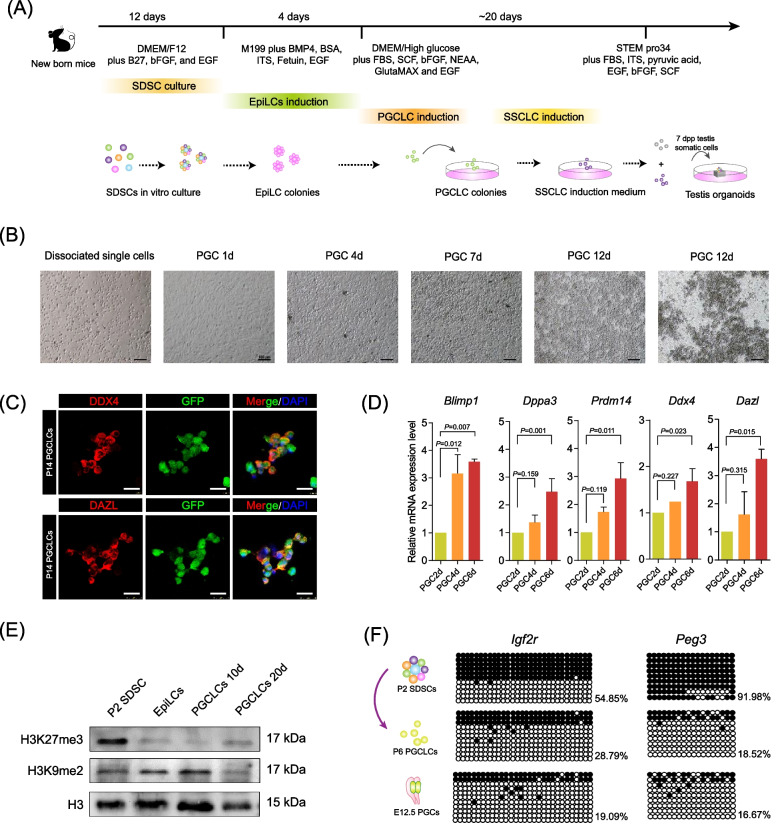
PGCLC differentiation from pDFPs. **A** Scheme showing PGCLC and SSCLC induction from SDSCs. **B** Representative morphology of PGCLCs generated from pDFPs at increasing culture times. Pictures were taken using an eclipse microscope (10x, Nikon TE2000, Japan). Scale bars, 100 μm. **C** Expression of the germ cell marker DDX4 and DAZL in day 6 eGFP positive pDFP-derived PGCLCs. Pictures were taken using an confocal microscope (20x, Leica, TCS SP5 II, Germany). Scale bars, 25 μm. **D** Expression of genes proper of specified PGCs by day 2, 4, 6 pDFP-derived PGCLCs. **E** Western blotting analysis of H3K27me3 and H3K9me2 expression during PGCLCs differentiation from pDFPs. **F** Bisulfite sequencing of DMRs of the imprinted genes *Igf2r* and *Peg3* in P2 SDSCs, day 6 pDFP-derived PGCLCs and E12.5 PGCs

We next investigated the germline potential of pDFPs and designed an optimized culture strategy to generate primordial germ cell-like cells (PGCLCs) from these cells. In order to efficiently generate PGCLCs from pDFPs we established a different protocol from those previously published using SDSCs [7, 8, 32]. In general, differentiation of PGCLCs from stem cells involves two main steps, induction of epiblast-like cells (EpiLCs) followed by PGCLC specification (Fig. 4A). On this basis in the present paper, eGFP-pDFPs were dissociated into single-cell pellets and were subjected to EpiLC induction under floating conditions in the presence of various concentrations of bone morphogenetic protein 4 (BMP4) (Additional file 3: Fig. S3A). The results showed that 30 ng/ml BMP4 promoted the formation of the largest eGFP-colonies both on day 1 and day 3 of culture (Additional file 3: Fig. S3B). These colonies can be considered bona fide EpiLCs since after real-time RT-PCR analyses they were shown to express a high level of the epiblast marker genes *Dnmt3a*, *Dnmt3b* and *Wnt3a* [7] (Additional file 3: Fig. S3C).

eGFP labelled EpiLCs were next dissociated into single cells and seeded onto MEF cells for PGCLC specification in the induction medium containing 30 ng/ml bone morphogenetic protein 4 (BMP4), 40 ng/ml stem cell factor (SCF), 40 ng/ml bFGF and 20 ng/ml EGF (Fig. 4B). After 4 days of differentiation, eGFP-positive round shiny cells could be observed on the surface of the feeder cells (Additional file 3: Fig. S3D). In subsequent days, these cells proliferated rapidly and gradually gave rise to floating clusters that stained positively for the two canonical late PGC markers DDX4 (also known as VASA) and DAZL [40] (Fig. 4C). The identity of these cells as a mixed population of early and late PGCLCs was further confirmed by the increased expression of the germ cell marker genes including *Ddx4*, *Dazl*, *Blimp1* (also known as *Prdm1*), *Dppa3* (also known as *Stella*), and *Prdm14* during prolonged in vitro culture (Fig. 4D).

The proper histone modifications and epigenetic reprogramming of PGCs are crucial for germ cell lineage specification [41], we then investigated whether histone modifications and epigenetic reprogramming proceeded properly in PGCLCs derived from pDFPs. Western blotting results showed that both H3 lysine 27 trimethylation (H3K27me3) and histone H3 lysine 9 dimethylation (H3K9me2) undergo progressive erasure and increase, respectively, from pDFPs to 10d PGCLCs showing a tendency to increase (H3K27me3) and erasure (H3K9me2) in 20d PGCLCs resembling the changes occurring in specified PGCs in vivo (Fig. 4E). The bisulfite sequencing of the differentially methylated regions (DMRs) of the imprinted *Igf2r* and *Peg3* loci showed lower levels of DNA methylation in P14 PGCLCs compared with P2 pDFPs, consistent with DNA methylation erasure typical of late PGCs in vivo. This process, however, appeared less efficient in PGCLCs than in their in vivo counterparts (Fig. 4F).

### Generation of SSCLCs from PGCLCs within testicular organoids

We then tested whether it was possible to induce pDFP-derived PGCLCs into spermatogonial stem cell-like cells (SSCLCs) as recently described from embryonic stem cells (ESCs) or induced pluripotent stem cells (iPSCs)-derived PGCLCs [21]. To this aim, we first changed the PGC induction medium with the SSC induction medium formulated by Ishikura et al., and mixed the eGFP-PGCLCs with somatic cells obtained from prepubertal testis to generate testicular organoids as described [21] with some modifications (see details in the Materials and Methods section).

**Fig. 5:**
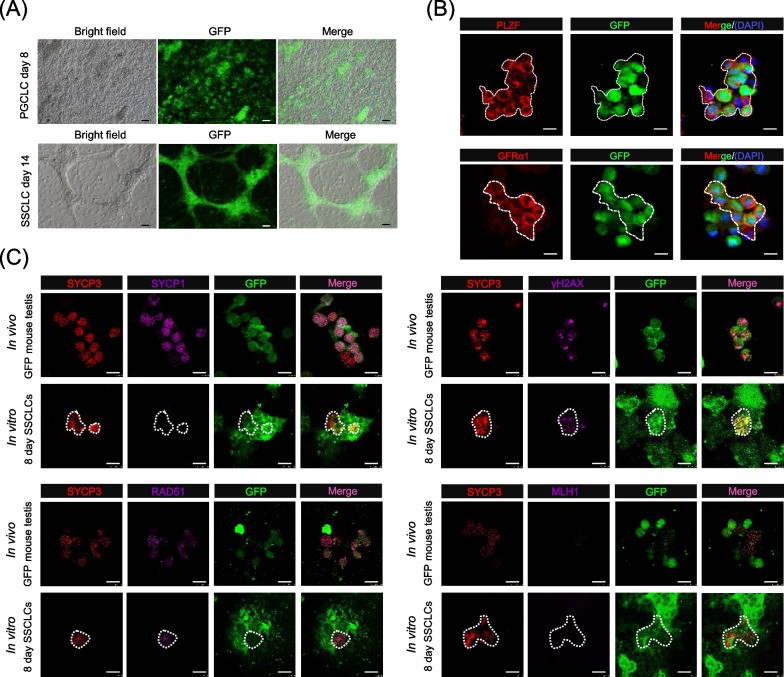
Induction of SSCLCs from pDFP-derived PGCLCs. **A** Representative pictures of day 7 and 14 aggregates of eGFP-PGCLCs and somatic testicular cells; at 14 days, testicular organoids showing structures resembling testis cords containing eGFP-cells are recognizable. Pictures were taken using an eclipse microscope (10x, Nikon TE2000, Japan). Scale bars, 50 μm. **B** Expression of the canonical SSC marker PLZF and GFRα1 in eGFP-cells isolated from testicular organoids after 14 days of culture. Pictures were taken using a confocal microscope (20x, Leica, TCS SP5 II, Germany). Scale bars, 20 μm. **C** Meiotic chromosome spreads of testicular tissues from 8 dpp testes of CAG/eGFP mice and testicular organoids containing eGFP-SSCLCs after 8 days of culture stained with antibodies against meiotic proteins; note that positive staining for SYCP3, γH2AX, and RAD51 was observed in all samples while positive cells for SYCP1 and MLH1 were observed only in the control samples from 8 dpp testes of CAG/eGFP mice. Pictures were taken using a confocal microscope (20x, Leica, TCS SP5 II, Germany). Scale bars, 20 μm

Beginning at day 4 of culture, cordonal structures resembling seminiferous cords containing eGFP-positive cells were observed (Fig. 5A). After dissociation of these structures at 14 days of culture, immunofluorescence staining for the canonical spermatogonia stem cell (SSC) proteins PLZF transcription repressor and GDNF receptor GFRa1 [42, 43], showed a significant number of double-positive eGFP-positive cells (Fig. 5B), thus suggesting their SSCLC identity. Finally, we investigated if these cells were able to enter meiosis within the cordonal structures. To this aim, the testicular organoid tissues were dissociated after 2, 4, 5, and 8 days of culture (Additional file 3: Fig. S4), and chromosome spreads were then performed to investigate whether these SSCLCs can initiate meiotic programs under in vitro conditions. Noteworthy, entering into meiosis of pDPFs-derived SSCLCs was further supported by chromosome spreads of testicular organoid tissues cultured for 8 days showing eGFP cells IF positive, in addition to SYCP3, and the two meiotic prophase proteins γH2AX and RAD51 (Fig. 5C) [31]. However, eGFP positive cells stained for the meiotic synapse protein SYCP1 and the recombination protein MLH1 were not found.

## Discussion

The main results achieved in the present paper were the identification of the cellular origin of the SDSCs from the skin of newborn mice and their efficient differentiation into cells of the male germ cell line.

Since in vitro isolation of multipotent stem cells from skin in early 2000 [11–14], the cells able to give rise to SDSCs have remained elusive. These cells were reported to reside in the dermis and, in principle, could be members of one or more of the stem cell populations residing in the hair follicles (bulge, dermal papillae and dermal sheath) and skin dermal layer, this latter including papillary, reticular and hypodermal fibroblast progenitors [33]. The lack of conserved lineage markers of these stem cells and the dynamics of the differentiation processes in which they are involved complicated discovering the origin of SDSCs. In the present paper, this question was solved using scRNA-seq and related bioinformatics analyses. It was revealed that SDSCs mainly consist of pDFPs, one of the three fibroblast precursors originating from multipotent dermal fibroblasts in the skin [33].

As reported in the introduction, in vitro SDSCs can be induced with distinct protocols to give rise to neuroectodermal and mesodermal progenies, including glia, adipocytes, neurons, smooth muscle cells, and, surprisingly, to cells of the germ cell lineage such as PGCLCs and OLCs [5]. In this regard, we have reported that it was possible to generate OLCs from HFSCs, one of the putative sources of SDSCs residing in the bulge region of the hair follicle and of epithelial derivation (see above), obtained from vibrissa hair follicles of 7 dpp mice [6]. We were, therefore, quite surprised to find that the scRNA-seq indicated pDFPs as the main members of SDSCs generated from the skin of mice of the same age. This suggests heterogenicity of SDSC spheres possibly with different cellular origins and prevalence of pDFPs when whole skin is used for their generation.

If SDSCs of distinct cellular origin also possess different GCLC potential remains to be determined. So far, however, the efficiency of the SDSC differentiation process into PGCLCs and OLCs and the quality of the GCLCs generated have been quite modest. As matter of fact, only mouse ESCs or iPSCs have been reported to generate germ cells able to complete spermatogenesis and oogenesis and give rise to functional gametes in vitro [3, 4]. The pluripotency of these stem cells and the induction to the EpiLC phenotype, seem two crucial conditions to generate male or female PGCLCs that are inducible into functional gametes. In any case, the production of functional sperm and oocytes in vitro requires inserting PGCCLs within reconstituted testes or ovaries [3, 44, 45]. Although SDSC-derived pDFPs do not possess pluripotency, supposing that they are probably less ‘biased’ than other ADSCs, we decided to investigate if subjected to a protocol of transient early mesodermal phenotype induction as that used for ESCs and iPSCs they were able to generate PGCLCs competent to differentiate into SSCs which has not previously been reported. For this last step, we utilized reconstituted testes to recapitulate SSC niche using a recently described three-dimensional multilayer model [20]. The results were promising since SDSC-derived pDFPs following induction into bona fide EpiLCs were efficiently specified into PGCLCs, following by cultured under SSC conditions, these PGCLCs generated SSCLCs which inserted within the reconstituted testicular organoids and appeared to be able to initiate meiosis. However, the generated SSCLCs showed a limited capacity of meiosis initiation. A previous study by Ishikura et al. [21], reported that abnormal epigenetic reprogramming is a limiting factor controlling the successful generation of spermatozoa from pluripotent stem cell-derived GCLCs. In this regard, the bisulfite sequencing analysis indicated that PGCLCs generated from SDSC-derived pDFPs possessed higher methylation levels at the DMRs of the imprinted *Igf2r* and *Peg3* loci compared with their in vivo PGC counterparts, thus suggesting that proper DNA demethylation of specific loci is a key regulatory factor controlling meiotic initiation and progression. This supports the notion that the epigenome of GCLCs derived from ASCs is a key indicator of their capability to correctly enter into meiosis. How establishing correct epigenetic reprogramming in ASC-derived GCLCs remains a crucial task for future studies. Key challenges ahead include of course reconstituting the entire spermatogenesis in vitro and in vivo from these GCLCs, but promising results have been already obtained using mouse ESCs [4].

## Conclusions

Here, by utilizing scRNA seq, we demonstrated that SDSCs derived from multipotent papillary dermal fibroblast progenitors (pDFPs) residing in the dermal layer show germline potential in vitro, and EGF signaling is pivotal for the capability of these progenitors to proliferate and form large colonies in vitro*.* More importantly, we established an optimized protocol to efficiently generate primordial germ cell-like cells (PGCLCs) from SDSCs. Together, these findings here lay the foundation for future generating functional gametes from adult stem cells.

### Supplementary Information

**Additional file 1: Table S1.** Antibodies used in the present study.

**Additional file 2: Table S2.** Primers used in the present study.

**Additional file 3: Fig. S1.** In vitro propagation of SDSCs and quality control of scRNA seq datasets. **A** Morphology of eGFP-SDSC spheres formation under the bright and fluorescent field. Pictures were taken using an eclipse microscope (10x, Nikon TE2000, Japan). Scale bars, 50 μm. **B** Quality matrices of two scRNA-seq datasets revealed by CellRanger. **C** Comparison of the number of genes detected in each cell (nFeature_RNA), the total number of molecules detected within a cell (nCount_RNA), and the percentage of mitochondria RNA in different datasets. **D** Representative marker expression in the UMAP plot. **Fig. S2.** Gene function enrichment comparison of different SDSC clusters. **A** Heatmap comparing the top enriched GO terms in different cell clusters. **B** Circos plot representations showing the overlapped GO terms and overlapped genes between different cell clusters. **Fig. S3.** EpiLC and PGCLC induction. **A** Effects of different concentrations of BMP4 on the formation of EpiLC colonies. Pictures were taken using an eclipse microscope (10x, Nikon TE2000, Japan). Scale bars, 100 μm. **B** Statistical comparison of the diameter of EpiLC colonies exposed to different concentrations of BMP4 on day 1 and day 3 of culture. **C** Expression of the epiblast Dnmt3a, Dnmt3b, and Wnt3a genes during EpiLCs induction revealed by RT-PCR. **D** Representative bright-field and fluorescent field image of day 4 eGFP-PGCLCs. Pictures were taken using an eclipse microscope (10x, Nikon TE2000, Japan). Scale bars, 100 μm. **Fig. S4.** Scheme of the testicular organoid setup.

## Data Availability

scRNA seq data of SDSCs are retrieved from our previous published dataset and the data are deposited in the NCBI GEO databases (https://www.ncbi.nlm.nih.gov/geo/) under Accession Number: GSE131498. scRNA seq data of SDSCs are deposited in the GSA database (https://bigd.big.ac.cn/) under Accession Number: CRA005534.

## Abbreviations

SDSCs: Skin-derived stem cells
scRNA-seq: Single-cell RNA sequencing
pDFPs: Papillary dermal fibroblast progenitors
EGF: Epidermal growth factor
PGCLCs: Primordial germ cell-like cells
SSCLCs: Spermatogonial stem cell like cells
EpiLCs: Epiblast cell like cells
ESCs: Embryonic stem cells
iPSCs: Induced pluripotent stem cells
ASCs: Adult stem cells
GCLCs: Germ cell-like cells
EPSCs: Epidermal stem cells
M-S-Cs: Mesenchymal stem cells
OLCs: Oocyte-like cells
HFSCs: Hair follicle stem cells
MEF: Mouse embryonic fibroblast
IF: Immunofluorescence
GO: Gene ontology
UMAP: Uniform manifold approximation and projection
DFs: Dermal fibroblast
DMR: Differentially methylated regions
SSC: Spermatogonia stem cell
RA: Retinoic acid

## References

[1] Mouka A, Tachdjian G, Dupont J, Drevillon L, Tosca L. In vitro gamete differentiation from pluripotent stem cells as a promising therapy for infertility. Stem Cells Dev. 2016;25(7):509–21. 26873432 10.1089/scd.2015.0230

[2] Ge W, Chen C, De Felici M, Shen W. In vitro differentiation of germ cells from stem cells: a comparison between primordial germ cells and in vitro derived primordial germ cell-like cells. Cell Death Dis. 2015;6(10):e1906. 26469955 10.1038/cddis.2015.265 PMC4632295

[3] Hikabe O, Hamazaki N, Nagamatsu G, Obata Y, Hirao Y, Hamada N, et al. Reconstitution in vitro of the entire cycle of the mouse female germ line. Nature. 2016;539(7628):299–303. 27750280 10.1038/nature20104

[4] Ishikura Y, Ohta H, Sato T, Murase Y, Yabuta Y, Kojima Y, et al. In vitro reconstitution of the whole male germ-cell development from mouse pluripotent stem cells. Cell Stem Cell. 2021;28:2167–79. 34496297 10.1016/j.stem.2021.08.005

[5] Ge W, Cheng SF, Dyce PW, De Felici M, Shen W. Skin-derived stem cells as a source of primordial germ cell- and oocyte-like cells. Cell Death Dis. 2016;7(11):e2471. 27831564 10.1038/cddis.2016.366 PMC5260893

[6] Sun YC, Ge W, Lai FN, Zhang RQ, Wang JJ, Cheng SF, et al. Oocyte-like cells induced from CD34-positive mouse hair follicle stem cells in vitro. J Genet Genom. 2017;44(8):405–7. 10.1016/j.jgg.2017.08.001 28844672

[7] Sun R, Sun YC, Ge W, Tan H, Cheng SF, Yin S, et al. The crucial role of Activin A on the formation of primordial germ cell-like cells from skin-derived stem cells in vitro. Cell Cycle. 2015;14(19):3016–29. 26406115 10.1080/15384101.2015.1078031 PMC4825550

[8] Dyce PW, Wen L, Li J. In vitro germline potential of stem cells derived from fetal porcine skin. Nat Cell Biol. 2006;8(4):384–90. 16565707 10.1038/ncb1388

[9] Linher K, Dyce P, Li J. Primordial germ cell-like cells differentiated in vitro from skin-derived stem cells. PLoS ONE. 2009;4(12):e8263. 20011593 10.1371/journal.pone.0008263 PMC2788220

[10] Danner S, Kajahn J, Geismann C, Klink E, Kruse C. Derivation of oocyte-like cells from a clonal pancreatic stem cell line. Mol Hum Reprod. 2007;13(1):11–20. 17114208 10.1093/molehr/gal096

[11] Toma JG, Akhavan M, Fernandes KJ, Barnabe-Heider F, Sadikot A, Kaplan DR, et al. Isolation of multipotent adult stem cells from the dermis of mammalian skin. Nat Cell Biol. 2001;3(9):778–84. 11533656 10.1038/ncb0901-778

[12] Dyce PW, Zhu H, Craig J, Li J. Stem cells with multilineage potential derived from porcine skin. Biochem Biophys Res Commun. 2004;316(3):651–8. 15033449 10.1016/j.bbrc.2004.02.093

[13] Jinno H, Morozova O, Jones KL, Biernaskie JA, Paris M, Hosokawa R, et al. Convergent genesis of an adult neural crest-like dermal stem cell from distinct developmental origins. Stem Cells. 2010;28(11):2027–40. 20848654 10.1002/stem.525 PMC3087810

[14] Rodrigues RM, De Kock J, Branson S, Vinken M, Meganathan K, Chaudhari U, et al. Human skin-derived stem cells as a novel cell source for in vitro hepatotoxicity screening of pharmaceuticals. Stem Cells Dev. 2014;23(1):44–55. 23952781 10.1089/scd.2013.0157 PMC3870603

[15] Dyce PW, Liu J, Tayade C, Kidder GM, Betts DH, Li J. In vitro and in vivo germ line potential of stem cells derived from newborn mouse skin. PLoS ONE. 2011;6(5):e20339. 21629667 10.1371/journal.pone.0020339 PMC3101249

[16] Park BW, Pan B, Toms D, Huynh E, Byun JH, Lee YM, et al. Ovarian-cell-like cells from skin stem cells restored estradiol production and estrus cycling in ovariectomized mice. Stem Cells Dev. 2014;23(14):1647–58. 24593690 10.1089/scd.2014.0029 PMC4086351

[17] Ge W, Ma HG, Cheng SF, Sun YC, Sun LL, Sun XF, et al. Differentiation of early germ cells from human skin-derived stem cells without exogenous gene integration. Sci Rep. 2015;5:13822. 26347377 10.1038/srep13822 PMC4561906

[18] Ge W, Tan SJ, Wang SH, Li L, Sun XF, Shen W, et al. Single-cell transcriptome profiling reveals dermal and epithelial cell fate decisions during embryonic hair follicle development. Theranostics. 2020;10(17):7581–98. 32685006 10.7150/thno.44306 PMC7359078

[19] Ge W, Zhang W, Zhang Y, Zheng Y, Li F, Wang S, et al. A single-cell transcriptome atlas of cashmere goat hair follicle morphogenesis. Genom Proteom Bioinform. 2021;19:437–51. 10.1016/j.gpb.2021.07.003 PMC8864196 34534715

[20] Alves-Lopes JP, Soder O, Stukenborg JB. Testicular organoid generation by a novel in vitro three-layer gradient system. Biomaterials. 2017;130:76–89. 28364632 10.1016/j.biomaterials.2017.03.025

[21] Ishikura Y, Yabuta Y, Ohta H, Hayashi K, Nakamura T, Okamoto I, et al. In vitro derivation and propagation of spermatogonial stem cell activity from mouse pluripotent stem cells. Cell Rep. 2016;17(10):2789–804. 27926879 10.1016/j.celrep.2016.11.026

[22] Fu XF, Yang F, Cheng SF, Feng YN, Li L, Dyce PW, et al. The epigenetic modifications and the anterior to posterior characterization of meiotic entry during mouse oogenesis. Histochem Cell Biol. 2017;148(1):61–72. 28235998 10.1007/s00418-017-1545-9

[23] Chao HH, Zhang XF, Chen B, Pan B, Zhang LJ, Li L, et al. Bisphenol A exposure modifies methylation of imprinted genes in mouse oocytes via the estrogen receptor signaling pathway. Histochem Cell Biol. 2012;137(2):249–59. 22131059 10.1007/s00418-011-0894-z

[24] Lucifero D, Mertineit C, Clarke HJ, Bestor TH, Trasler JM. Methylation dynamics of imprinted genes in mouse germ cells. Genomics. 2002;79(4):530–8. 11944985 10.1006/geno.2002.6732

[25] Ge W, Wang JJ, Zhang RQ, Tan SJ, Zhang FL, Liu WX, et al. Dissecting the initiation of female meiosis in the mouse at single-cell resolution. Cell Mol Life Sci. 2021;78(2):695–713. 32367190 10.1007/s00018-020-03533-8 PMC11072979

[26] Stuart T, Butler A, Hoffman P, Hafemeister C, Papalexi E, Mauck WM 3rd, et al. Comprehensive integration of single-cell data. Cell. 2019;177(7):1888–902. 31178118 10.1016/j.cell.2019.05.031 PMC6687398

[27] La Manno G, Soldatov R, Zeisel A, Braun E, Hochgerner H, Petukhov V, et al. RNA velocity of single cells. Nature. 2018;560(7719):494–8. 30089906 10.1038/s41586-018-0414-6 PMC6130801

[28] Bergen V, Lange M, Peidli S, Wolf FA, Theis FJ. Generalizing RNA velocity to transient cell states through dynamical modeling. Nat Biotechnol. 2020;38(12):1408–14. 32747759 10.1038/s41587-020-0591-3

[29] Street K, Risso D, Fletcher RB, Das D, Ngai J, Yosef N, et al. Slingshot: cell lineage and pseudotime inference for single-cell transcriptomics. BMC Genom. 2018;19(1):477. 10.1186/s12864-018-4772-0 PMC6007078 29914354

[30] Frede J, Anand P, Sotudeh N, Pinto RA, Nair MS, Stuart H, et al. Dynamic transcriptional reprogramming leads to immunotherapeutic vulnerabilities in myeloma. Nat Cell Biol. 2021;23(11):1199–211. 34675390 10.1038/s41556-021-00766-y PMC8764878

[31] Zhou Y, Zhou B, Pache L, Chang M, Khodabakhshi AH, Tanaseichuk O, et al. Metascape provides a biologist-oriented resource for the analysis of systems-level datasets. Nat Commun. 2019;10(1):1523. 30944313 10.1038/s41467-019-09234-6 PMC6447622

[32] Shen W, Park BW, Toms D, Li J. Midkine promotes proliferation of primordial germ cells by inhibiting the expression of the deleted in azoospermia-like gene. Endocrinology. 2012;153(7):3482–92. 22564978 10.1210/en.2011-1456

[33] Driskell RR, Lichtenberger BM, Hoste E, Kretzschmar K, Simons BD, Charalambous M, et al. Distinct fibroblast lineages determine dermal architecture in skin development and repair. Nature. 2013;504(7479):277–81. 24336287 10.1038/nature12783 PMC3868929

[34] Phan QM, Fine GM, Salz L, Herrera GG, Wildman B, Driskell IM, et al. Lef1 expression in fibroblasts maintains developmental potential in adult skin to regenerate wounds. eLife. 2020;9:e60066. 32990218 10.7554/eLife.60066 PMC7524549

[35] Lee CS, Bhaduri A, Mah A, Johnson WL, Ungewickell A, Aros CJ, et al. Recurrent point mutations in the kinetochore gene KNSTRN in cutaneous squamous cell carcinoma. Nat Genet. 2014;46(10):1060–2. 25194279 10.1038/ng.3091 PMC4324615

[36] Wang Y, Wen J, Zhang W. MIIP, a cytoskeleton regulator that blocks cell migration and invasion, delays mitosis, and suppresses tumorogenesis. Curr Protein Pept Sci. 2011;12(1):68–73. 21190522 10.2174/138920311795659434

[37] Voss M, Paterson J, Kelsall IR, Martin-Granados C, Hastie CJ, Peggie MW, et al. Ppm1E is an in cellulo AMP-activated protein kinase phosphatase. Cell Signal. 2011;23(1):114–24. 20801214 10.1016/j.cellsig.2010.08.010

[38] Rezza A, Wang Z, Sennett R, Qiao W, Wang D, Heitman N, et al. Signaling networks among stem cell precursors, transit-amplifying progenitors, and their niche in developing hair follicles. Cell Rep. 2016;14(12):3001–18. 27009580 10.1016/j.celrep.2016.02.078 PMC4826467

[39] Sennett R, Wang Z, Rezza A, Grisanti L, Roitershtein N, Sicchio C, et al. An integrated transcriptome atlas of embryonic hair follicle progenitors, their niche, and the developing skin. Dev Cell. 2015;34(5):577–91. 26256211 10.1016/j.devcel.2015.06.023 PMC4573840

[40] Nicholls PK, Schorle H, Naqvi S, Hu YC, Fan Y, Carmell MA, et al. Mammalian germ cells are determined after PGC colonization of the nascent gonad. Proc Natl Acad Sci USA. 2019;116(51):25677–87. 31754036 10.1073/pnas.1910733116 PMC6925976

[41] De Felici M. Nuclear reprogramming in mouse primordial germ cells: epigenetic contribution. Stem cells international. 2011;2011:425863. 21969835 10.4061/2011/425863 PMC3182379

[42] Buaas FW, Kirsh AL, Sharma M, McLean DJ, Morris JL, Griswold MD, et al. Plzf is required in adult male germ cells for stem cell self-renewal. Nat Genet. 2004;36(6):647–52. 15156142 10.1038/ng1366

[43] Costoya JA, Hobbs RM, Barna M, Cattoretti G, Manova K, Sukhwani M, et al. Essential role of Plzf in maintenance of spermatogonial stem cells. Nat Genet. 2004;36(6):653–9. 15156143 10.1038/ng1367

[44] Ishikura Y, Ohta H, Sato T, Murase Y, Yabuta Y, Kojima Y, et al. In vitro reconstitution of the whole male germ-cell development from mouse pluripotent stem cells. Cell Stem Cell. 2021;28(12):2167–79. 34496297 10.1016/j.stem.2021.08.005

[45] Yoshino T, Suzuki T, Nagamatsu G, Yabukami H, Ikegaya M, Kishima M, et al. Generation of ovarian follicles from mouse pluripotent stem cells. Science. 2021;373(6552):eabe0237. 34437124 10.1126/science.abe0237

