# Correction: Murine skin-derived multipotent papillary dermal fibroblast progenitors show germline potential in vitro

**DOI:** 10.1186/s13287-024-03838-6

**Published:** 2024-07-23

**Authors:** Wei Ge, Yuan-Chao Sun, Tian Qiao, Hai-Xia Liu, Tao-Ran He, Jun-Jie Wang, Chun-Lei Chen, Shun-Feng Cheng, Paul W. Dyce, Massimo De Felici, Wei Shen

**Affiliations:** 1 College of Life Sciences, Key Laboratory of Animal Reproduction and Biotechnology in Universities of Shandong, Qingdao Agricultural University, Qingdao, 266109 China; 2 Department of Animal Sciences, Auburn University, Auburn, AL 36849 USA; 3 Department of Biomedicine and Prevention, University of Rome Tor Vergata, 00133 Rome, Italy

**Correction: Stem Cell Research & Therapy (2023) 14:17** 10.1186/s13287-023-03243-5

The authors note that during the preparation of the manuscript, the track plot included the expression trends of 14 genes (with Tk1 and Pclaf repeated twice), but only 13 gene symbols were labeled, resulting in a mismatch and repeat of the trackplot with the image on the right. This error occurred during the typesetting process of the original figures. The authors have corrected the annotations in Fig. 2C as shown ahead in this correction article, apologise for the error, and confirm that the overall results and conclusions are not affected by this change.

**Fig. 2:**
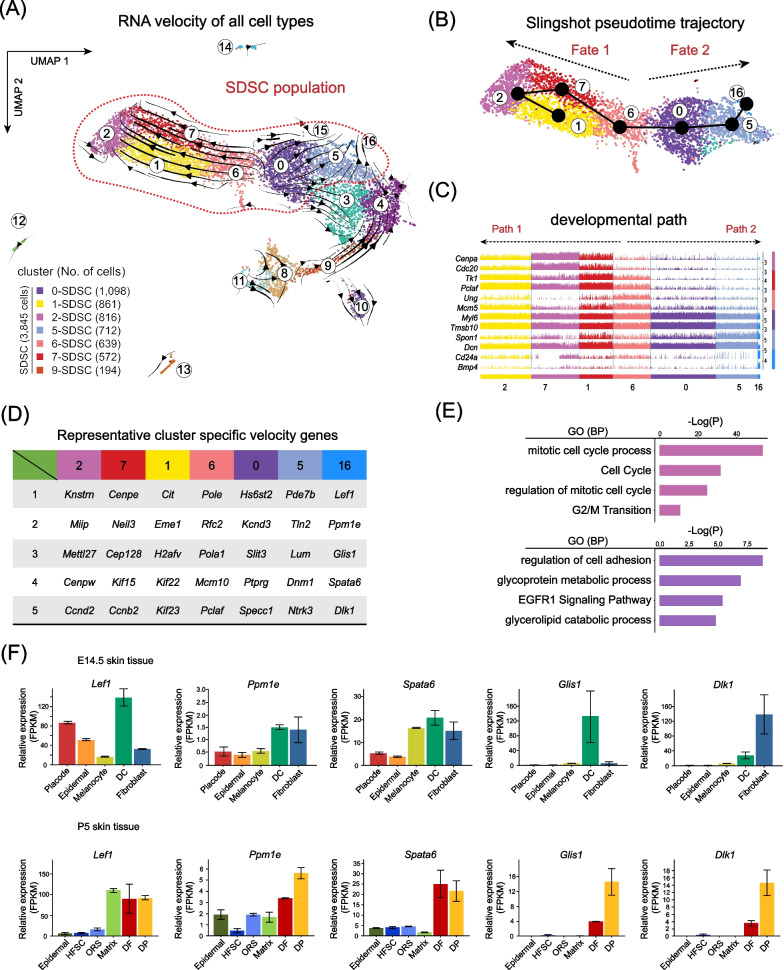
Combined RNA velocity and trajectory inference unveil the cellular origin of SDSCs. **A** Projection of RNA velocity vectors in the UMAP plot. **B** Slingshot infers the pseudotime trajectory in the P2 SDSCs. **C** Expression of cell fate 1 and cell fate 2 representative marker genes along pseudotime trajectories. **D** Expression of top 5 cell cluster-specifc RNA velocity genes; genes were ranked by their roles in driving the velocity trajectories. **E** GO enrichment analysis of top 100 RNA velocity genes in the end state of cell fate 1 and 2. **F** Expression of top 5 cell fate 2 RNA velocity genes in the skin of E14.5 foetuses and 5 dpp newborn skin

