# Supplementary material for: Murine skin-derived multipotent papillary dermal fibroblast progenitors show germline potential in vitro
Source: Stem Cell Res Ther. 2023 Feb 3;14:17. doi: 10.1186/s13287-023-03243-5 (PMC9898921; doi:10.1186/s13287-023-03243-5)

Supplementary figure 1

(A)

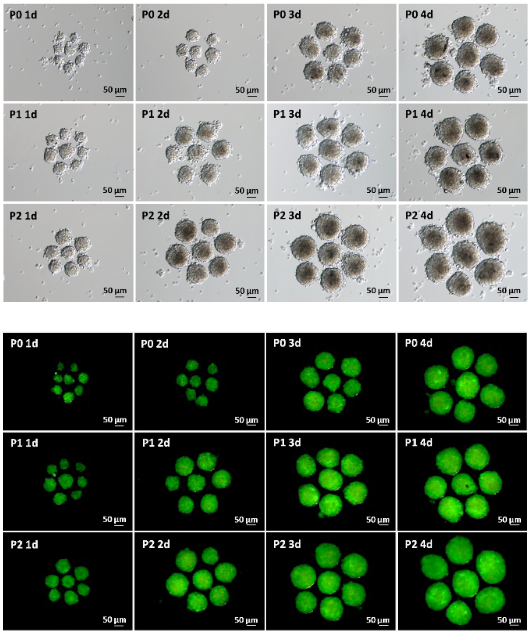

(B)

Sample information	0 dpp skin	SDSC
Estimated number of cells	4000	4000
Percentage of valid barcodes	97.6%	97.7%
Mean number of reads per cell	132,231	160,183
Median number of genes per cell	2,424	4,709
Total number of genes detected	19,435	18,864
Percentage of reads mapped to genome	94.0%	90.3%

(C)

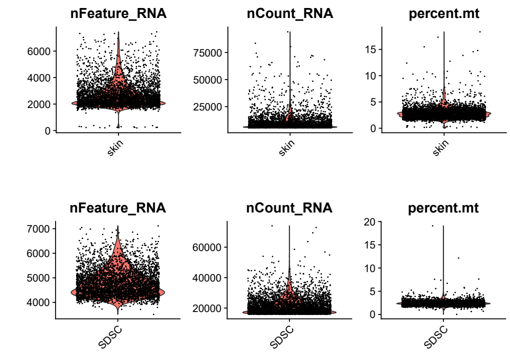

(D)

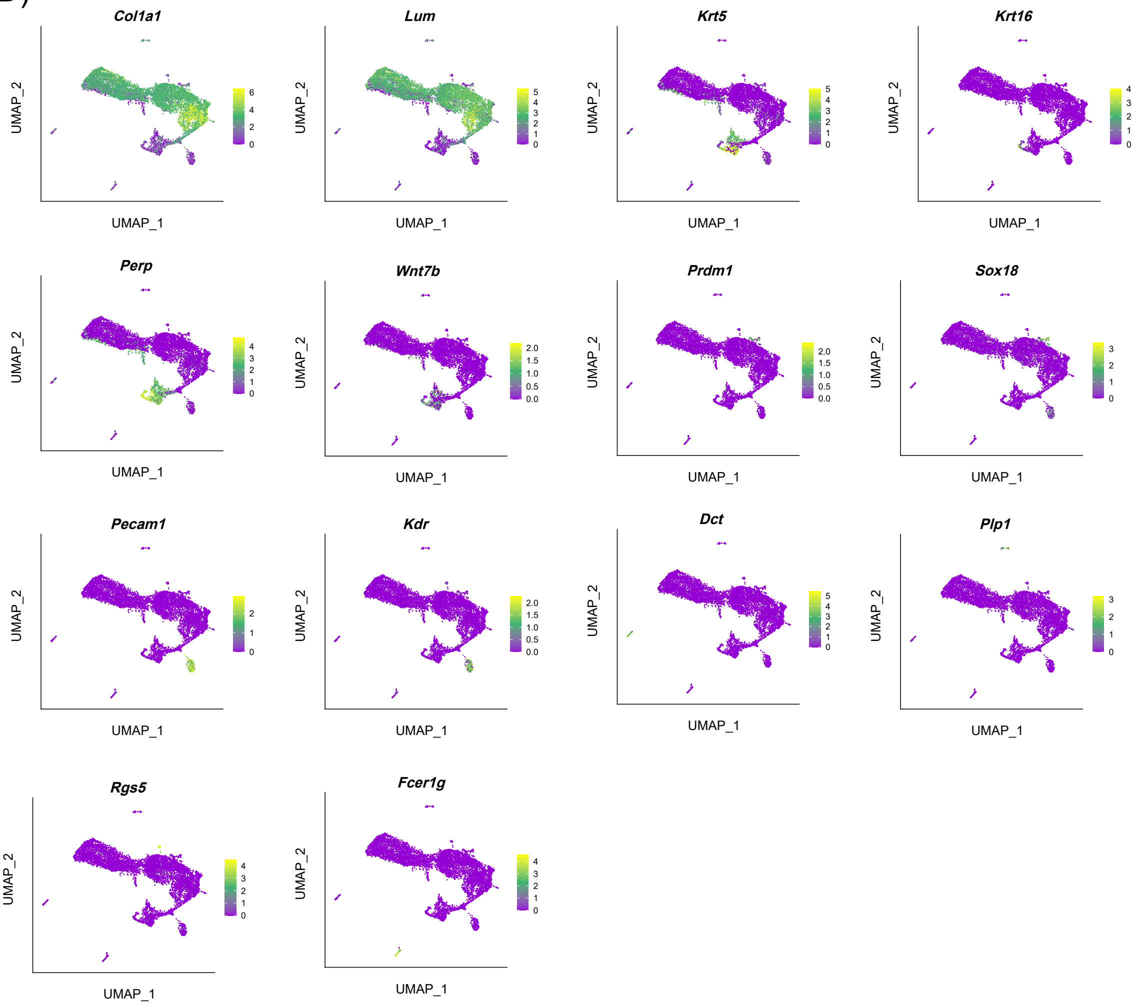

Supplementary figure 2

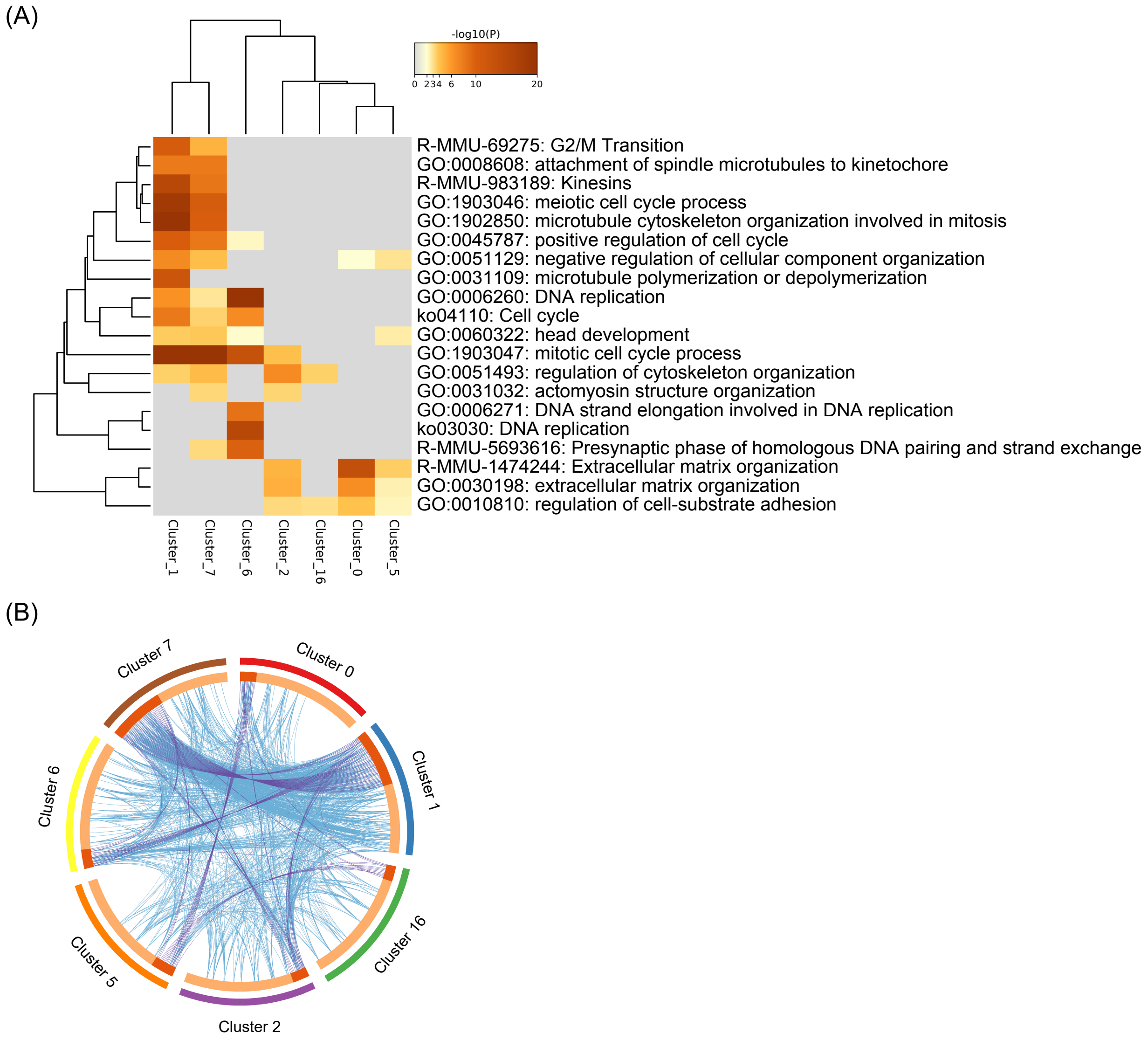

Supplementary figure 3

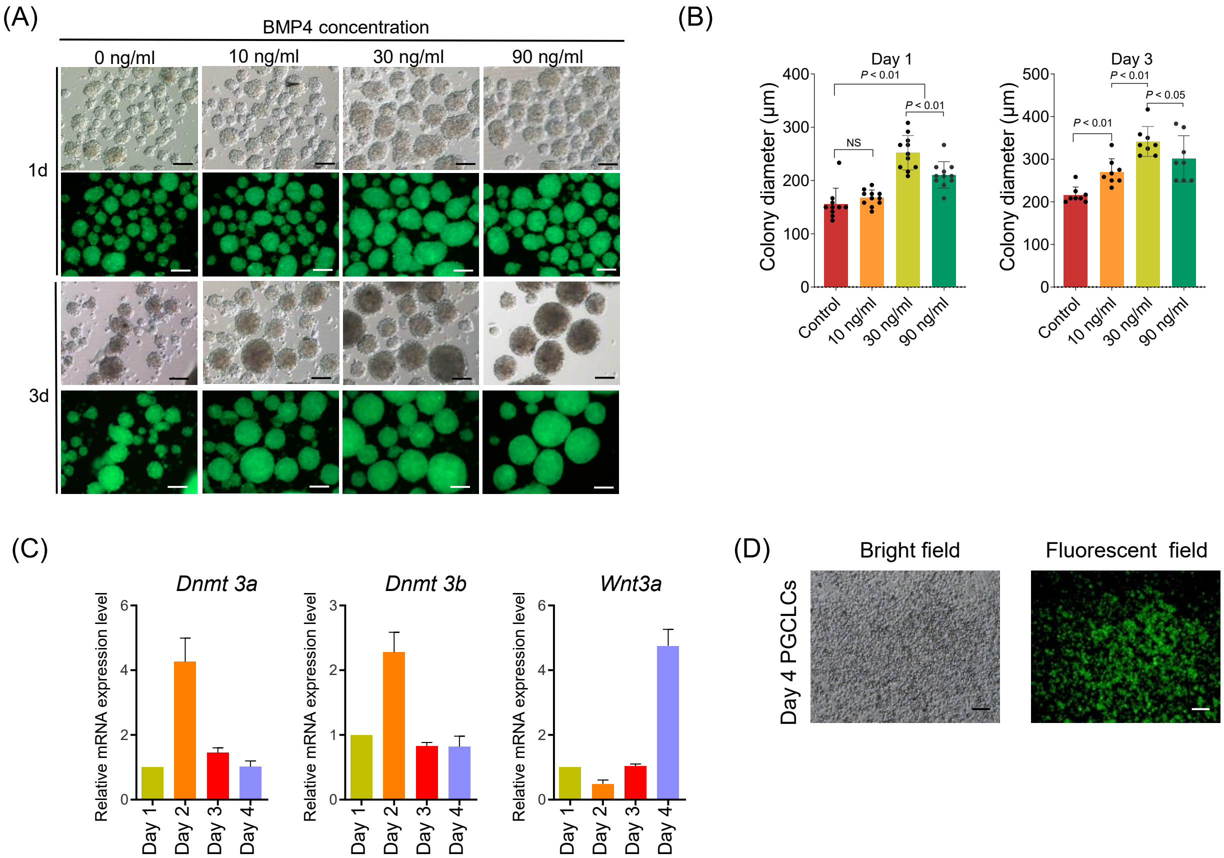

Supplementary figure 4

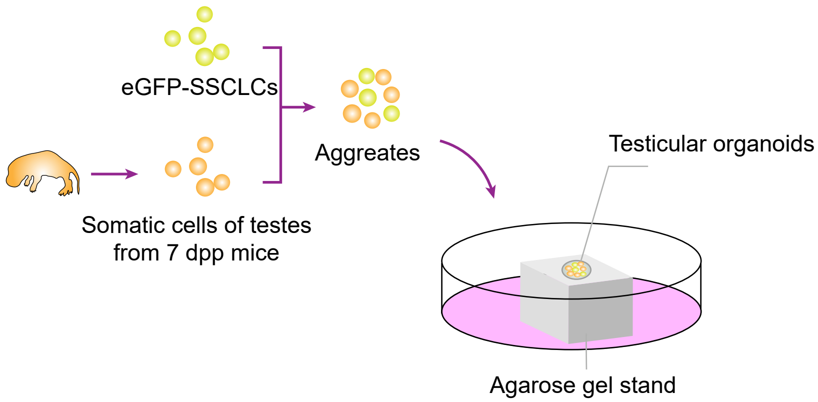

Supplement: Supplementary file 3 — Additional file 3: Fig. S1. In vitro propagation of SDSCs and quality control of scRNA seq datasets. A Morphology of eGFP-SDSC spheres formation under the bright and fluorescent field. Pictures were taken using an eclipse microscope (10x, Nikon TE2000, Japan). Scale bars, 50 μm. B Quality matrices of two scRNA-seq datasets revealed by CellRanger. C Comparison of the number of genes detected in each cell (nFeature_RNA), the total number of molecules detected within a cell (nCount_RNA), and the percentage of mitochondria RNA in different datasets. D Representative marker expression in the UMAP plot. Fig. S2. Gene function enrichment comparison of different SDSC clusters. A Heatmap comparing the top enriched GO terms in different cell clusters. B Circos plot representations showing the overlapped GO terms and overlapped genes between different cell clusters. Fig. S3. EpiLC and PGCLC induction. A Effects of different concentrations of BMP4 on the formation of EpiLC colonies. Pictures were taken using an eclipse microscope (10x, Nikon TE2000, Japan). Scale bars, 100 μm. B Statistical comparison of the diameter of EpiLC colonies exposed to different concentrations of BMP4 on day 1 and day 3 of culture. C Expression of the epiblast Dnmt3a, Dnmt3b, and Wnt3a genes during EpiLCs induction revealed by RT-PCR. D Representative bright-field and fluorescent field image of day 4 eGFP-PGCLCs. Pictures were taken using an eclipse microscope (10x, Nikon TE2000, Japan). Scale bars, 100 μm. Fig. S4. Scheme of the testicular organoid setup. [file 13287_2023_3243_MOESM3_ESM.pdf]
